# A comparative genomics study of the microbiome and freshwater resistome in Southern Pantanal

**DOI:** 10.3389/fgene.2024.1352801

**Published:** 2024-04-18

**Authors:** André R. de Oliveira, Bárbara de Toledo Rós, Rodrigo Jardim, Nelson Kotowski, Adriana de Barros, Ricardo H. G. Pereira, Nalvo Franco Almeida, Alberto M. R. Dávila

**Affiliations:** ^1^ Laboratório de Biologia Computacional e Sistemas, Instituto Oswaldo Cruz, Rio de Janeiro, Brazil; ^2^ Universidade Federal do Mato Grosso do Sul, Campo Grande, Brazil; ^3^ Universidade Federal do Mato Grosso do Sul, Aquidauana, Brazil

**Keywords:** resistome, metagenomics, microbiome, pantanal, freshwater

## Abstract

This study explores the resistome and bacterial diversity of two small lakes in the Southern Pantanal, one in Aquidauana sub-region, close to a farm, and one in Abobral sub-region, an environmentally preserved area. *Shotgun* metagenomic sequencing data from water column samples collected near and far from the floating macrophyte *Eichhornia crassipes* were used. The Abobral small lake exhibited the highest diversity and abundance of antibiotic resistance genes (ARGs), antibiotic resistance classes (ARGCs), phylum, and genus. RPOB2 and its resistance class, multidrug resistance, were the most abundant ARG and ARGC, respectively. Pseudomonadota was the dominant phylum across all sites, and *Streptomyces* was the most abundant genus considering all sites.

## 1 Introduction

Antibiotic resistance is emerging as a significant global public health issue due to the swift rise of resistant bacteria and the concurrent decline in new drugs entering the market ​​(Serwecińska, 2020)​​. While resistance is a natural phenomenon in microbial communities, where it serves as a form of competition ​​(Frost et al., 2018)​​, its effects can be amplified in environments with high antibiotic concentrations. These environments include livestock farms ​(Qian et al., 2018)​​, aquacultures ​​(Preena et al., 2020)​​, hospital effluents ​​(Hassoun-Kheir et al., 2020)​​, and wastewater treatment plants ​(Raza et al., 2022)​​. Bacteria possess horizontal gene transfer mechanisms (integrons, MGE, and plastids) that facilitate the spread of antibiotic resistance genes (ARGs) within the community ​​(Sun et al., 2019)​​. Consequently, a pathogenic species can develop resistance to a specific antibiotic without direct exposure to it.

Although antibiotic resistance in pathogenic bacteria is well-studied, these bacteria represent only a small fraction of the microbial community ​(Doron and Gorbach, 2008)​​. Therefore, our understanding of the antibiotic-resistance genes in non-pathogenic bacteria, particularly those inhabiting rivers, lakes, soils, and oceans, remains limited. This gap in knowledge is due to the difficulty in cultivating these bacteria using current protocols. Metagenomics, which allows for the analysis of a microbial community through sequencing of environmental genetic material without the need for cultivation ​(Doron and Gorbach, 2008)​​, has emerged as a promising solution.

Given the central role these microorganisms play in biogeochemical processes, studies on this topic have increased. Understanding the resistome of non-culturable bacterial communities is crucial for identifying potential gene reservoirs that could contribute to the evolution and spread of antibiotic resistance ​(Fresia et al., 2019)​.​

The significance of biotic antibiotic removal mechanisms, often carried out by microorganisms, is highlighted in this context. These mechanisms are intertwined with various plant-based processes, a phenomenon known as phytoremediation. Certain plants have demonstrated the ability to eliminate and tolerate high levels of antibiotics without experiencing toxic effects ​(Kurade et al., 2021; Polińska et al., 2021)​​. As a result, the microbial community present is shaped by the presence of plants and their impact on the rhizosphere, which in turn affects antibiotic resistance.

Our study is centered on the Pantanal Sul Matogrossense, one of the world’s largest wetland ecosystems ​​(Assine, 2015)​​. This biome covers parts of Brazil (78%), Bolivia (18%), and Paraguay (4%). Despite its recognition, it has been significantly impacted by livestock activities, which often involve the use of large quantities of antibiotics ​​(Ferrante and Fearnside, 2022)​. This region, abundant in water, serves as an efficient medium for the spread of Mobile Genetic Elements (MGE). The vast water systems in the Pantanal enhance the potential for ARGs to disseminate widely ​(Aminov and Mackie, 2007; Baquero et al., 2008)​​. Hence, the examination of this region’s resistome is of paramount importance from both a public health and academic perspective.

This study is set to explore the resistome and taxonomy of filtered water samples from two small lakes in Pantanal. One small lake is in a farm area, the other in a reserve, where samples were taken both near and far from the *Eichhornia crassipes* (Mart.) Solms macrophyte. We’ll analyze sequencing data, looking into the identity and diversity of the Antibiotic Resistance Genes (ARGs), and also classify the samples taxonomically. This is the first time a metagenomic approach is being used to study the bacterial resistome of this area.

## 2 Methods

### 2.1 Sampling

**FIGURE 1:**
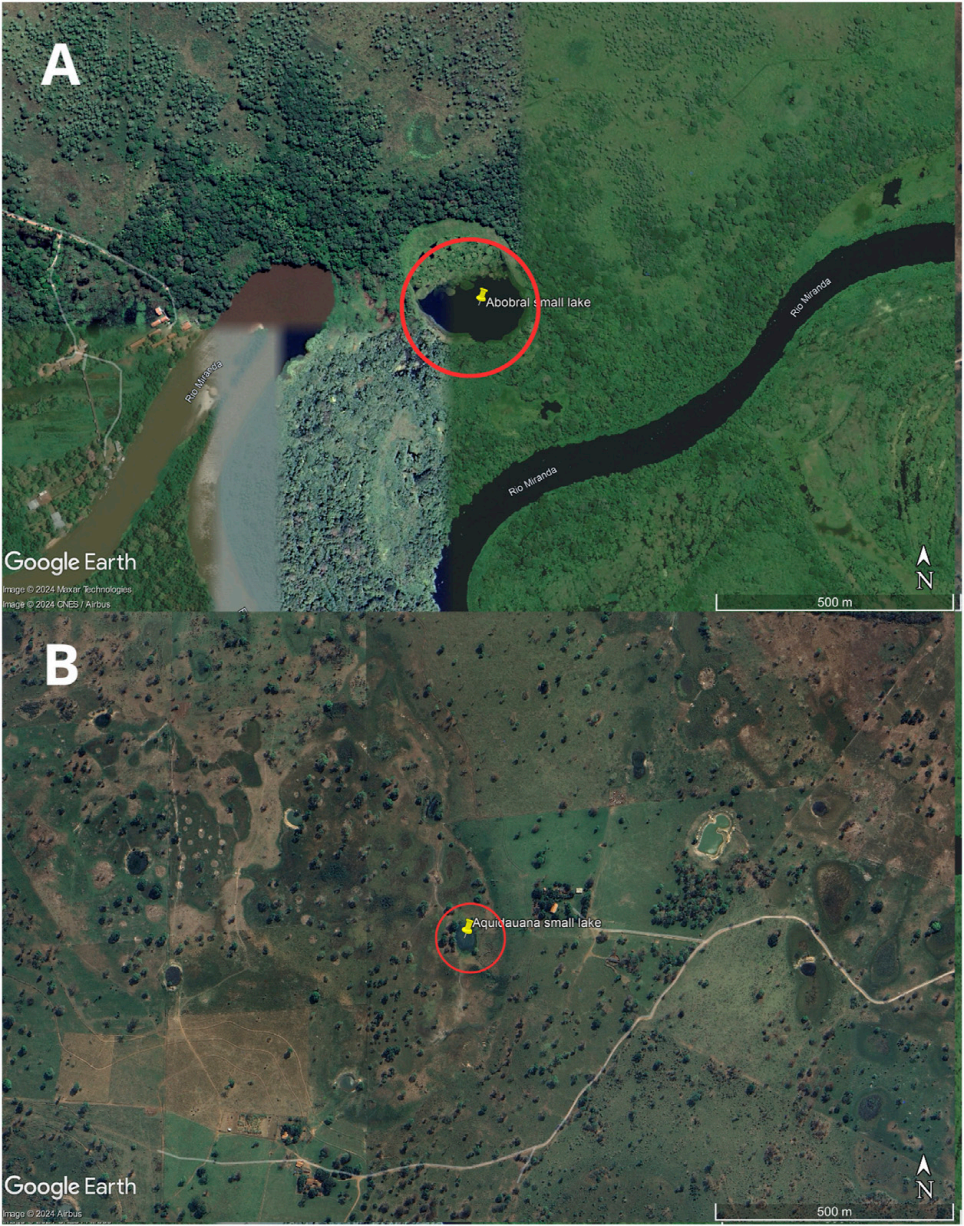
Map of the sample collection location. The small lakes are highlighted by a red circle. Image **(A)** is from Abobral and **(B)** is from Aquidauana.

**FIGURE 2:**
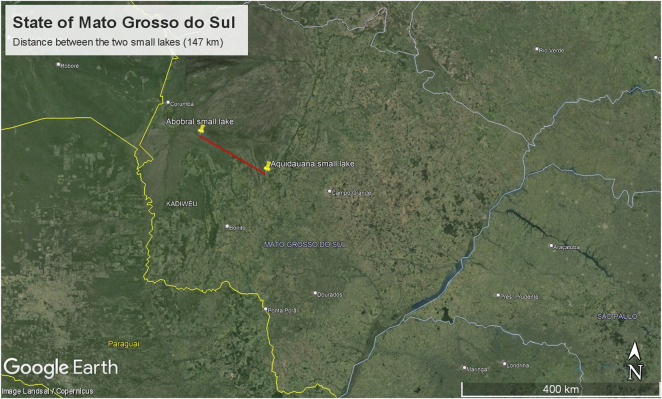
Map displaying the distance between the small lakes.

The sampling site comprised two small lakes located in the Pantanal Sul Matogrossense: one within an environmental reserve unit in the Pantanal de Abobral subregion (Figure 1A), municipality of Corumbá (19°34′35″S 57°00′46″W), and the other in a farm region in the Pantanal de Aquidauana subregion (Figure 1B), municipality of Aquidauana (20°12′30″S 55°46′29″W), 147 km away from each other (Figure 2). In each small lake, water column samples were collected near the floating macrophyte (*Eichhornia crassipes*) and at a distance of 10 m from it. Access to genetic samples was properly registered at Brazilian SisGen under the code: AB3AE33. The sequencing files were submitted to the NCBI Bioproject under the code PRJNA1078255.

For clarity and objectivity, the sample from Abobral near the macrophyte will be referred to as “Site 1”, Abobral distant from the macrophyte as “Site 2”, Aquidauana near the macrophyte as “Site 3”, and Aquidauana distant from the macrophyte as “Site 4”. Each sample consisted of 10 L of water, collected in autoclaved bottles and subsequently stored at 4°C.

In the laboratory, the water samples were filtered through 1.2µm, 0.8µm, and 0.45 µm membranes, but only the data from the last one were used for this work. After the DNA extraction with QIAGEN DNEasy PowerWater Kit, the genetic material extracted from each of the 10 L bottled water was sent to the Sequencing Platform of the Histocompatibility and Cryopreservation Laboratory of the State University of Rio de Janeiro (UERJ) for a *shotgun* sequencing on the Illumina HiSeq-2500 platform. The sequenced data were then stored on the servers of the Laboratory of Computational Biology and Systems at Oswaldo Cruz Institute/Fiocruz and submitted to NCBI SRA (PRJNA1078255).

### 2.2 Data analysis

The sequencing data were processed using two sequence analysis tools: the Metawrap pipeline (version 1.3) ​(Uritskiy et al., 2018)​ and DeepArg (version 2.0) ​(Arango-Argoty et al., 2018)​. The Metawrap pipeline was utilized for the following steps: i) Quality verification of the sequences using FastQC, ii) Sequence cleaning with Trimmomatic, iii) Taxonomic inference using Kraken, and iv) Taxonomy visualization with Krona. Following the cleaning process conducted by Trimmomatic, the data were further analyzed by DeepArg. This allowed for the prediction of Antibiotic Resistance Genes (ARGs) and their corresponding Antibiotic Resistance Classes (ARGCs), following the classification scheme provided by the tool.

All statistical analyses were performed using the R programming language. The descriptive statistics, including all diversity indices, were computed using the Vegan package (version 2.15–1)​(Dixon, 2003)​ The graphics were generated with the ggplot2 package (version 3.4.4).

Due to the large volume of data, a selection criterion was established for the ARGs, ARGCs, phylum, and genus to be included in the graphical analysis. The criteria for inclusion were a relative abundance of 5% or higher for ARGs, ARGCs, and phylum in at least one sample site. For genus, the threshold was set at a relative abundance of 1% or higher. This adjustment was necessary as only two genera had a relative abundance higher than 5%. This approach ensured a manageable and representative subset of data for graphical analysis.

The inferential statistics involved testing for normality using the Shapiro-Wilk test, Lilliefors test, and QQ plot. Based on these normality tests, only non-parametric tests were appropriate for our data. Therefore, group comparisons were made using the Kruskal–Wallis test, followed by a *post hoc* Dunn’s test with Bonferroni correction.

These comparisons were performed on alpha and beta diversity indices for ARGs, ARGCs, and the diversity of phyla and genera. Given that the Abobral and Aquidauana small lakes do not have direct contact, and therefore, their communities are isolated from each other, beta diversity was analyzed only among locations that establish a habitat gradient (WHITTAKER, 1972), that is, in each isolated small lake, taking into account only the presence and absence of macrophyte. The bootstrap resampling process (R = 100) was used to enhance the reliability of the results. All tests were considered significant at *p* < 0.05.

## 3 Results

### 3.1 Diversity of ARGs and ARGCs

A total of 232 ARGs and 66 ARGCs were found adding up all sites. Among them, we were able to identify a total of 103 unique ARGs and 21 Antibiotic Resistance Gene Classes (ARGCs). Comparing all sites, Site one stood out with the most ARGs (74), ARGCs (19), and the highest number of reads (1,685). On the other hand, Site 3 had the least with 48 ARGs, 14 ARGCs, and 874 reads (Table 1).

**TABLE 1:** Number of ARGs, ARGCs, reads, phylum and genus found in each Site.

*Information*	*Site 1*	*Site 2*	*Site 3*	*Site 4*
ARGs	74	55	48	55
ARGCs	19	18	14	15
reads	1,685	1,399	874	974
phylum	26	24	22	26
genus	468	485	384	442

A comparative analysis between the small lakes revealed that Abobral’s small lake (Sites 1 and 2) had a greater diversity of unique ARGs (84) and ARGCs (21) than Aquidauana’s small lake (Sites 3 and 4), which had 69 ARGs and 17 ARGCs.

When considering the presence (Sites 1 and 3) and absence (Site 2 and 4) of the macrophyte, its presence seemed to slightly increase the number of different ARGs (82 vs. 79) but decrease the number of ARGCs (19 vs. 21). The co-occurrence of ARGs and ARGCs, whether together or isolated, is illustrated in Supplementary Figures S1 and S2. At least 24 ARGs and 11 known ARGCs were found to occur together in all sites.

**FIGURE 3:**
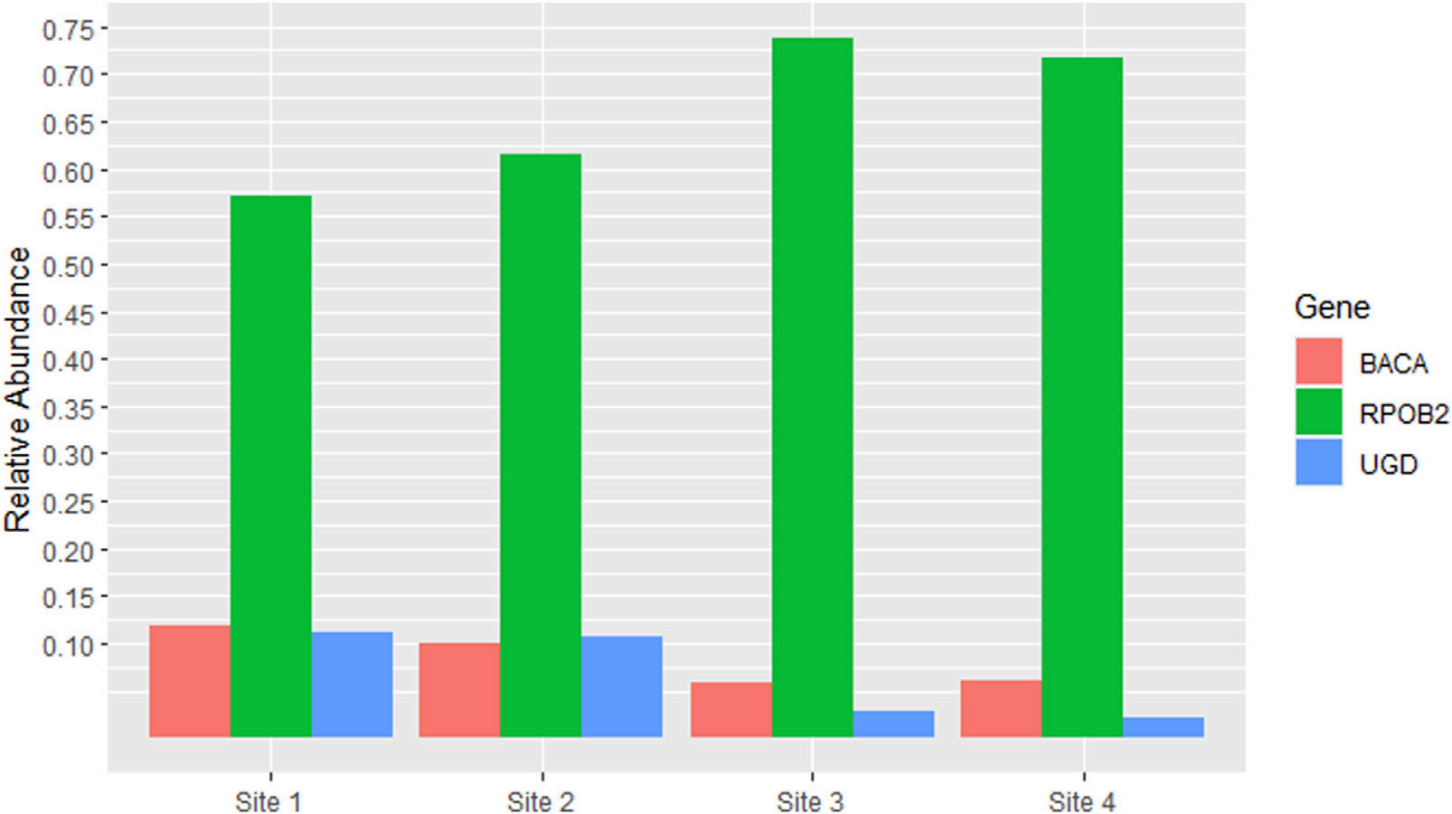
Antibiotic Resistance Genes (ARGs) which had relative abundance equal to or greater than 5% in at least one site. The *y*-axis represents the percentage of relative abundance (0.75 = 75%).

The most abundant ARG was RPOB2, accounting for more than half of the total reads in all samples, with Site 3 having the highest percentage (73.8%). Other ARGs that had high percentages compared to the others were BACA and UGD in Sites 1 and 2, both accounting for approximately 10% of the total reads in each site (Figure 3).

**FIGURE 4:**
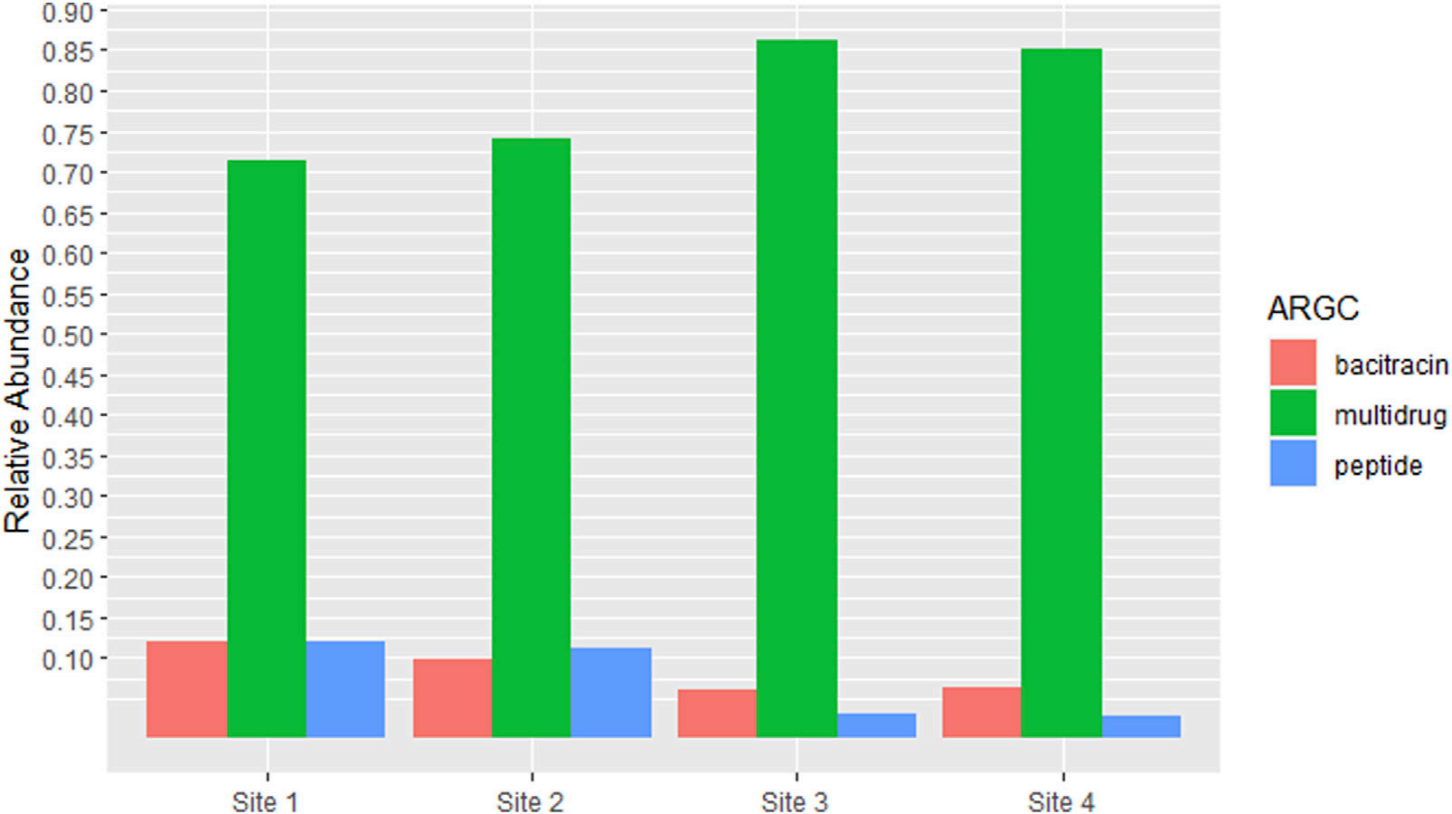
Antibiotic Resistance Classes (ARGCs) which had relative abundance equal to or greater than 5% in at least one site. The *y*-axis represents the percentage of relative abundance.

Given that RPOB2 was the most abundant ARG across all samples, its corresponding antibiotic resistance class, Multidrug Resistance, would consequently be the most prevalent. Bacitracin and Peptides come in sequence also reflecting the proportions of the genes BACA and UGD (Figure 4). The richness and abundance of all ARGs and ARGC present in each site can be found in Supplementary Figures S2 and S4.

A total of 98 phyla and 1779 genera were found adding up all sites. Among them, we were able to identify a total of 31 unique phyla and 917 genera. Comparing all sites, Site 1 and 4 stood out with the most unique phyla (26) and Site 2 with the most genera (485). On the other hand, Site 3 had the least with 22 phyla and 384 genera (Table 1).

Similarly to the ARGs and ARGCs comparative analysis, Abobral’s small lake (Sites 1 and 2) had a greater diversity of unique phyla (31) and genera (687) than Aquidauana’s small lake (Sites 3 and 4), which had 27 phyla and 576 genera. When considering the presence (Sites 1 and 3) and absence (Site 2 and 4) of the macrophyte, its presence seemed to also slightly increase the number of different phyla (32 vs. 29) but decrease the number of genera (653 vs. 700).

**FIGURE 5:**
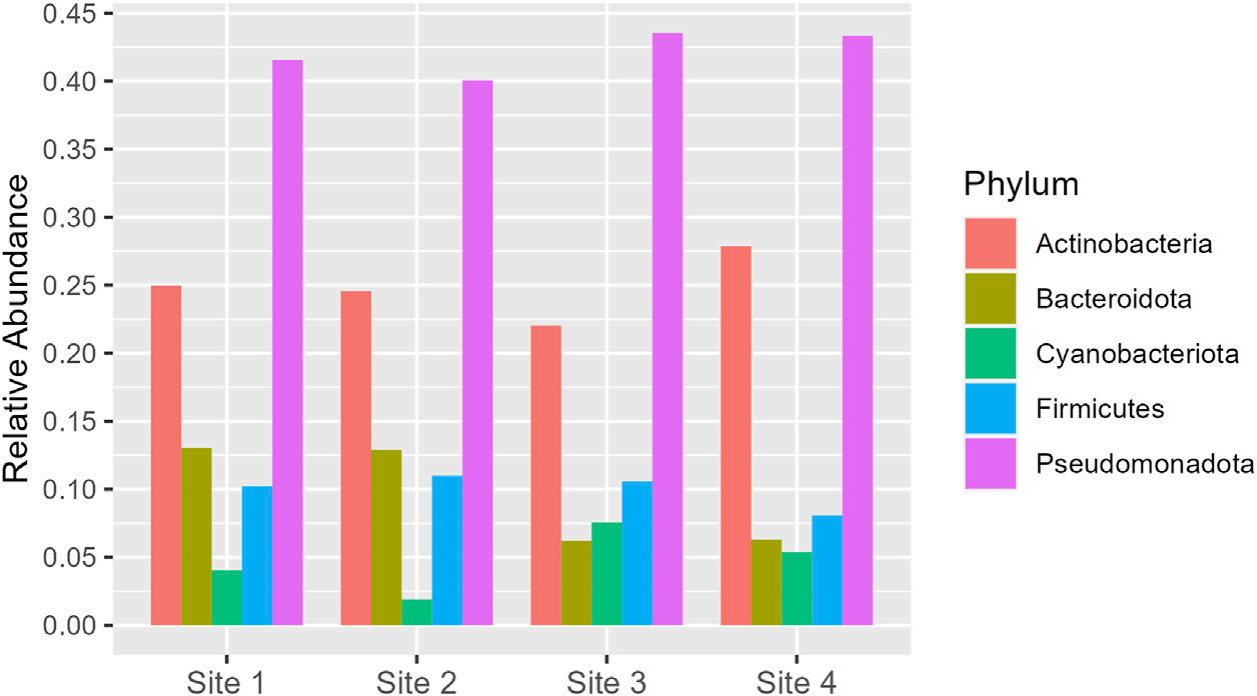
Phyla which had relative abundance equal to or greater than 5% in at least one site. The *y*-axis represents the percentage of relative abundance.

Pseudomonadota is the dominant phylum across all sites, with relative abundance varying from 0.40 to 0.43. Actinobacteria follows as the second most abundant, with a slight increase in proportion at Site 4 (0.28) compared to the range of 0.22–0.25 at the other sites. Bacteroidota and Firmicutes exhibit similar distributions, but Bacteroidota shows a notable decrease at Site 3 and Site 4 (around 0.06) compared to Site one and Site 2 (around 0.13). Cyanobacteriota, although the least abundant, show a significant increase at Site 3 (0.08) compared to Site 2 (0.02) (Figure 5). More information about all the phyla found in all sites can be seen in Supplementary Material (Supplementary Figure S5).

**FIGURE 6:**
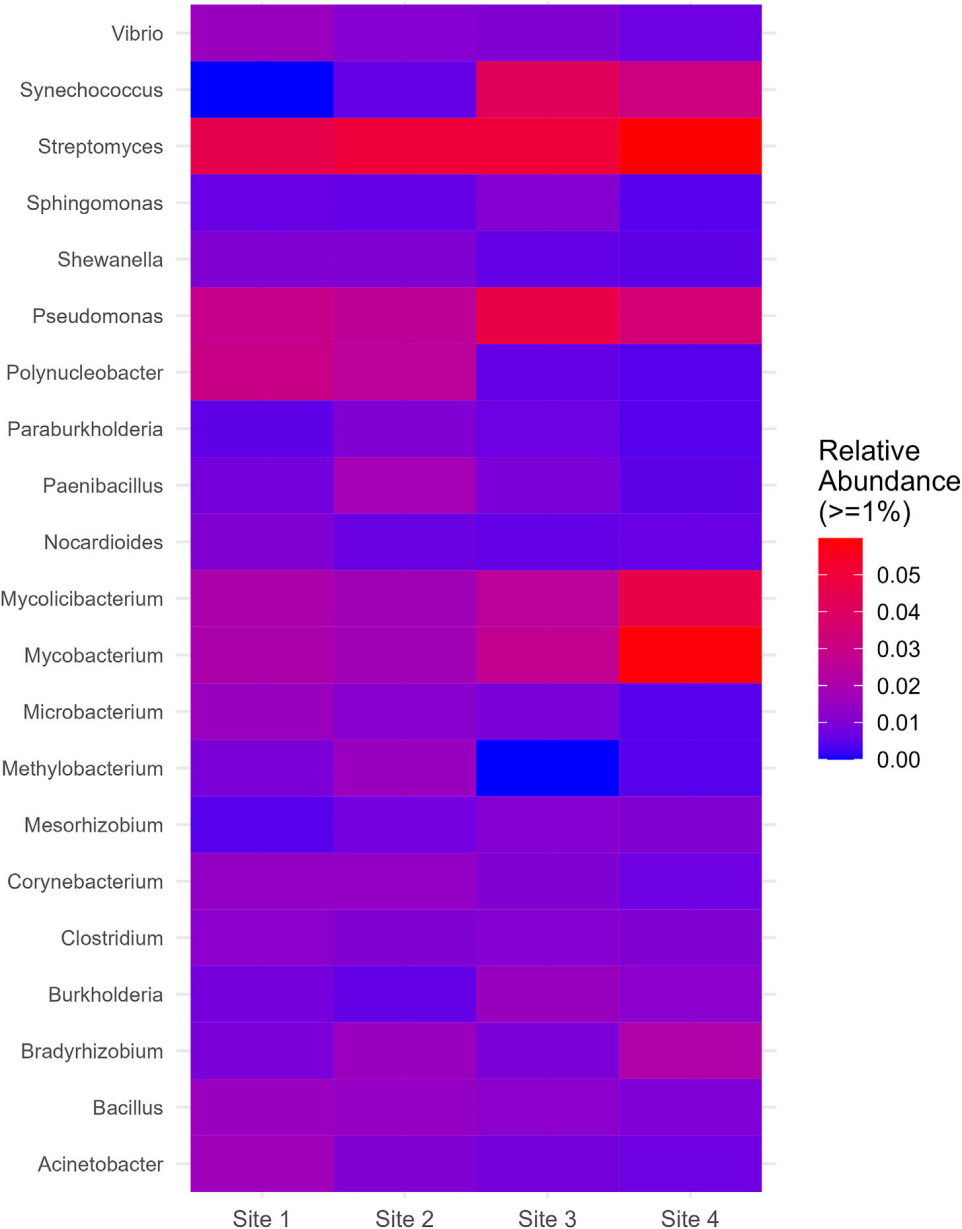
Genus which had relative abundance equal to greater than 1% in at least one site.

On the genus level, *Streptomyces* and *Pseudomonas* were relatively abundant across all sites and displayed a slight increase at Site 4 and Site 3, respectively. *Mycobacterium* and *Mycolicibacterium*, while exhibiting similar distributions, showed a significant increase at Site 4. Synechococcus, which was absent at Site 1, manifested a substantial increase at Site 3 and Site 4. *Polynucleobacter*, on the other hand, showed a significant decrease at Site 3 and Site 4. *Methylobacterium*, despite its low relative abundance at Site one and Site 2, was completely absent at Site 3 and appeared in lower proportions at Site 4 (Figure 6). More information about all the genera found in all sites can be seen in Supplementary Material (Supplementary Figure S6).

### 3.2 Diversity indexes

Both Simpson and Shannon indices were employed to assess the diversity for the following categories: ARGs, ARGCs, phylum, and genus. The results are listed in Tables 2, 3.

**TABLE 2:** Simpson Indexes for ARGs, ARGCs, phylum and genus.

*Simpson Index*	*Site 1*	*Site 2*	*Site 3*	*Site 4*
ARG	0.64	0.60	0.44	0.47
ARGC	0.46	0.42	0.25	0.26
phylum	0.74	0.74	0.74	0.71
genus	0.99	0.99	0.99	0.99

**TABLE 3:** Shannon Indexes for ARGs, ARGCs, phylum and genus.

*Shannon Index*	*Site 1*	*Site 2*	*Site 3*	*Site 4*
ARG	1.85	1.67	1.37	1.48
ARGC	0.99	0.94	0.63	0.69
phylum	1.67	1.74	1.75	1.68
genus	5.51	5.57	5.32	5.28

**FIGURE 7:**
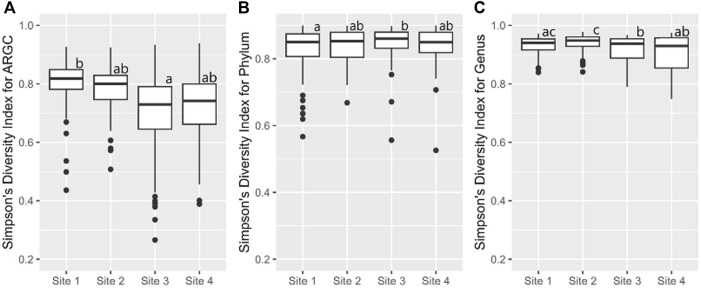
From left to right, bootstrap boxplots from the Simpson’s Index of ARGC **(A)**, phylum **(B)**, and genus **(C)** diversity of each site. Letters inside the graph represent Dunn’s test results.

Further statistical analysis revealed distinct differences in diversity across various sites. The Simpson index revealed key differences in ARGC diversity between Sites one and 3 (Figure 7A), phylum diversity between Sites 1 and 3 (Figure 7B), and genus diversity between Sites 2 and 3, and Sites 2 and 4 (Figure 7C).

**FIGURE 8:**
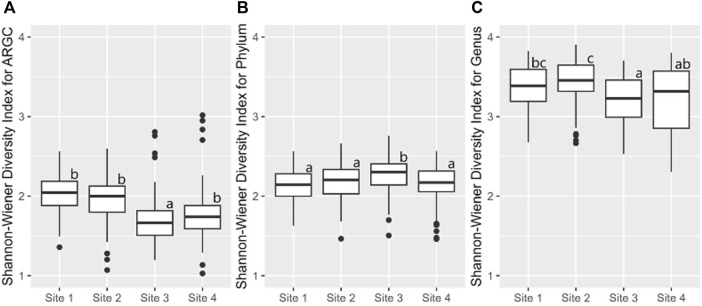
From left to right, bootstrap boxplots from the Shannon-Wiener Index of ARGC **(A)**, phylum **(B)**, and genus **(C)** diversity of each site. Letters inside the graph represent Dunn’s test results.

Building on this, the Shannon index identified additional disparities. In ARGC diversity, it showed differences between Sites 1, 2, and 3 (Figure 8A). For phylum diversity, it highlighted differences between Sites 2, 3, and 4 (Figure 8B). For genus diversity, it confirmed differences between Sites 1, 2, and 4 (Figure 8C). No significant differences were observed for ARGs in either index.

The beta diversity analysis, utilizing Bray Curtis and Sørensen dissimilarity indices, is listed in Table 4:

**TABLE 4:** Beta diversity indexes.

*Sites*	*ARGs*		*ARGCs*		*genus*		*phylum*	
	*Bray-Curtis Index*	*Sørensen Index*	*Bray-Curtis Index*	*Sørensen Index*	*Bray-Curtis Index*	*Sørensen Index*	*Bray-Curtis Index*	*Sørensen Index*
Site 1 vs. Site 2	0.096	0.448	0.047	0.135	0.343	0.440	0.055	0.240
Site 3 vs. Site 4	0.072	0.340	0.022	0.172	0.335	0.391	0.075	0.125

**FIGURE 9:**
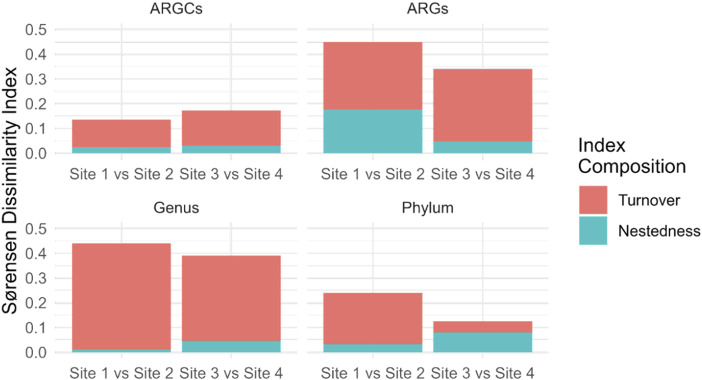
Sørensen Dissimilarity decomposition into Turnover and Nestedness for ARGs, ARGCs, genus and phylum between sites close and far from the macrophyte for each small lake (Site 1 vs. Site 2 and Site 3 vs. Site 4).

Further decomposition of the Sørensen index into turnover and nestedness components revealed noteworthy turnover. Sites 1 and 2 had the highest turnover for genus at 0.4295 and the lowest in phylum at 0.2083. Sites 3 and 4 had pronounced turnover, especially in ARGs at 0.2917 and phylum at 0.0455. Nestedness was observed to a lesser extent, emphasizing the distinct ecological compositions between the small lake sites (Figure 9).

**FIGURE 10:**
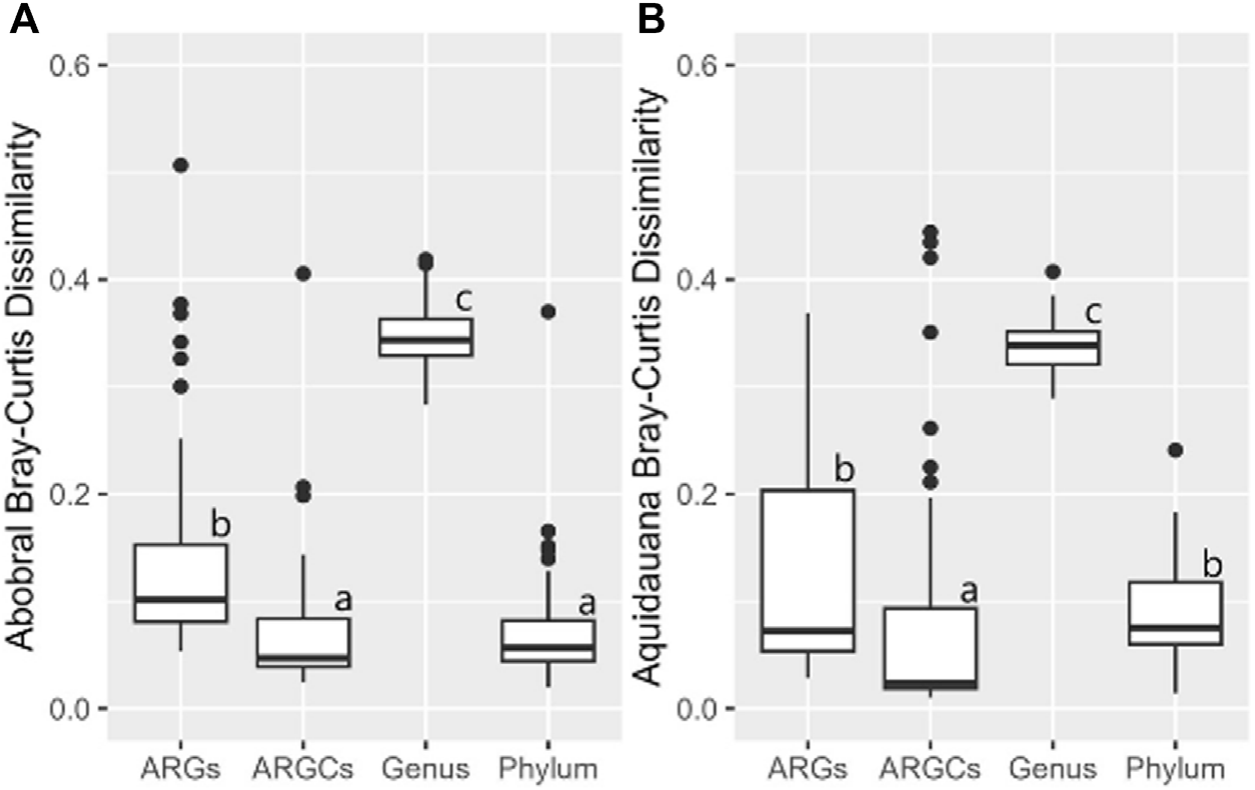
Bray-Curtis Dissimilarity for ARGs, ARGCs, genus, and phylum between sites close and far from the macrophyte for each small lake. **(A)** Site 1 vs Site 2, **(B)** Site 3 vs Site 4.

The inferential statistics were conducted to evaluate whether the diversity difference caused by the presence of macrophytes was significantly greater for one group compared to the others. In Abobral, only ARGC and phylum were not different from one another (Figure 10A). A similar pattern was noted in Aquidauana, where only ARGs and phylum showed no significant difference (Figure 10B).

## 4 Discussion

### 4.1 ARGs, ARGC and read numbers

Antibiotics and antibiotic resistance genes (ARGs) in freshwater environments are influenced by a myriad of factors, including soil type, macrophyte species, type of macrophyte, antibiotic concentration, water flow, microbiota, community dynamics, nutrients, pH, oxygenation, temperature, and the method of antibiotic introduction ​(Overton et al., 2023)​. While this study provides valuable insights into the resistome and taxonomy of the sampled sites, it is important to acknowledge that these biotic and abiotic parameters were not measured during the sample collection and could potentially explain the observed results.

In the present study a higher concentration of ARGs, ARGCs, and reads in a conserved area with no human activity compared to a farm region were found, suggesting that human activity may not always lead to an increase in ARGs in nearby bacterial communities. This could be potentially explained by the well-established fact that conserved areas have higher biodiversity in contrast to anthropized areas ​(McDonald et al., 2020; Glidden et al., 2021)​.

The significant presence of the RPOB2 gene in both Abobral and Aquidauana suggests the existence of a natural reservoir for this gene within the Pantanal region. The geographical extent of this reservoir warrants further investigation. This finding holds considerable interest for both public health and academic research.

The observed shift in the proportions of the ARGs RPOB2, BACA, UGD and their respective ARGCs within the Aquidauana may be attributed to a complex interplay of factors. While the small lake’s proximity to an agricultural region suggests that farming practices could have influenced these shifts, it is important to question this assumption. The use of antibiotics, heavy metals, and other agrochemicals, which can infiltrate local water bodies through various means, could have contributed to the loss of genetic diversity in this region ​(Holt, 2000; Schmitt et al., 2015)​.

However, it is crucial to conduct further studies to determine whether the observed decrease in biodiversity is a permanent or transient phenomenon, possibly due to a recent change in environmental conditions or farming practices. A study demonstrated that the introduction of an antibiotic to the microbiological community was initially harmful. However, from the second to the fifth week, the levels of bacterial activity returned to levels similar to those found in communities not exposed to the antibiotic ​(Weber et al., 2011)​. This observation further underscores the need for ongoing monitoring and research to fully understand the dynamics at play.

There were unique ARGs and ARGCs found exclusively in certain locations, and these did not appear in isolation but were always found together, suggesting a possible relationship or co-dependence (Supplementary Figure S1). Moreover, the presence of the macrophyte influenced the occurrence of certain ARGs and ARGCs, demonstrating that their presence was more influenced by the macrophyte rather than the small lake environment.

One specific group of clinically important genes found in all samples were the mcr-N genes. These genes are significant because they offer resistance to colistin, an antibiotic used as a last resort against super-resistant bacteria. Although they have already been found in remote places, such as Antarctica, any occurrence of this group of genes is important to be reported ​(Cuadrat et al., 2020)​.

### 4.2 Diversity indexes

In this study, we used two different indices to measure alfa diversity: the Shannon index and the Simpson index. Overall the Shannon index identified Abobral small lake with the higher biodiversity compared to Aquidauana. However, for the Simpson index, it was the opposite. This might seem contradictory at first, however, this is due to the different aspects of biodiversity these indices measure.

The Shannon index is sensitive to species richness, meaning it increases with the number of different species present. Conversely, the Simpson index emphasizes species evenness, meaning it increases when a few species are significantly more prevalent than others ​(Guiaşu and Guiaşu, 2003)​.

A study on the effects of simulated nitrogen deposition on soil microbial community diversity in a coastal wetland found that with increasing levels of nitrogen deposition, alpha diversity (Shannon and Simpson indices) decreased significantly (Lu et al., 2021). This decrease in diversity may be due to soil acidification resulting from long-term nitrogen deposition in the supersaturated state. Long-term nitrogen deposition may also decrease the available organic matter of soil microorganisms, reducing microbial activity and diversity. Another study found that the presence of certain pollutants, such as chromium, can also reduce alpha diversity ​(Wei et al., 2023)​. Although we do not have data regarding nutrient and contaminant concentrations, bodies of water near farms often have high concentrations of these nutrients and heavy metals due to agricultural practices.

On the contrary, studies conducted in a wastewater treatment plant and different types of soil found that alpha diversity was higher in areas with more pollution and human intervention ​(Ndlovu et al., 2016; Geng et al., 2020)​. The addition of Fe^2+^ was also found to increase microbial diversity ​(Song et al., 2016)​. These findings suggest that while certain conditions and substances can decrease alpha diversity, others can lead to an increase, highlighting the complex interplay of factors that influence microbial diversity.

The results of beta diversity indexes indicate that the influence of macrophytes on the diversity of the small lake ecosystem varies across different categories. The genus category showed the highest dissimilarity values across both indices and all sites, indicating that the genus-level diversity is most affected by the proximity to the macrophyte. This is expected given that genus was the category with the highest diversity of all, allowing for a greater variation.

In contrast, the ARGCs and phylum categories showed relatively lower dissimilarity values. This suggests that these categories are less influenced by the proximity to the macrophyte compared to the genus and ARGs categories.

The dissimilarity values were generally higher for the Abobral small lake (Site 1 vs. Site 2) compared to Aquidauana’s (Site 3 vs. Site 4) across all categories. This is also expected since the former exhibited greater diversity, allowing for more variation.

The decomposition of the Sørensen index into turnover and nestedness components provided a more nuanced understanding of the differences in microbial diversity between the sites. The results suggest that turnover is the dominant component of dissimilarity for all categories at both sites, indicating that the differences in diversity between the sites are primarily due to the replacement of species, rather than the presence of a subset of species at one site. In contrast, the nestedness component was relatively low for all categories, suggesting that the sites do not contain many species that are subsets of the species at other sites.

These findings emphasize the importance of considering multiple indexes when assessing biodiversity. Each index provides a different lens through which to view diversity, and together they offer a more comprehensive picture of the ecological structure of the sites.

### 4.3 Taxonomy

The abundance results found for the diversity of phyla and genera are consistent with the genetic diversity of the small lakes, with the Abobral small lake exhibiting greater diversity than Aquidauana.

The Pseudomonadota phylum (previous Proteobacteria), known for its high abundance in the majority of microbial communities, was, indeed, the most prevalent across all samples. This group plays a significant role in removing various pollutants, making it a crucial component of the microbial community. Its dominance in most systems can be attributed to its diverse metabolic capabilities and adaptability to different environmental conditions, which allow it to thrive in both polluted and unpolluted freshwater environments ​(Holt et al., 1984; Balows et al., 1992; Bergey, 1994; Topley, 2005)​.

In addition to its prevalence, this phylum of microorganisms plays a significant role in various environmental processes. For instance, the removal of antibiotics is largely attributed to the majority of functional microorganisms within this phylum ​(Alexandrino et al., 2017; Huang et al., 2017; Li et al., 2019; Shan et al., 2020)​. Additionally, the class *Deltaproteobacteria*, which is part of the Pseudomonadota phylum, contains most of the sulfate-reducing bacteria essential for heavy metal removal ​(Chen et al., 2021a; 2021b)​. The phylum is also involved in the removal of phosphorus, as indicated by research from ​(Si et al., 2018; 2019; Huang et al., 2020)​. Lastly, the role of the Pseudomonadota phylum in nitrogen removal from various wastewaters is well-documented, with genera such as *Nitrosomonas*, *Nitrobacter*, and *Nitrosospira* being associated with nitrification ​(Aguilar et al., 2019; Ajibade et al., 2021)​.

The *Pseudomonas* genus, a member of the Pseudomonadota phylum, is renowned for its adaptability and metabolic flexibility ​(He et al., 2004; Høiby et al., 2010)​. This genus is found in various environments ​(Özen and Ussery, 2012)​, demonstrating its resilience and ability to thrive even in polluted areas. Its metabolic versatility allows it to play a crucial role in the removal of different pollutants. *Pseudomonas* exhibits a remarkable capability for environmental remediation. It effectively absorbs phosphorus from wastewater, storing it internally as polyphosphate ​(Tian et al., 2017; Huang et al., 2020)​. This process not only aids in the purification of wastewater but also contributes to the recycling of this essential nutrient.

In addition to phosphorus absorption, *Pseudomonas* shows resistance to heavy metals, aiding in their extraction from the environment. This is achieved through the synthesis of extracellular substances that bind to these metals, thereby preventing their spread within the biofilm and offering protection to the cells from stress ​(Teitzel and Parsek, 2003; Giovanella et al., 2017)​.

*Pseudomonas* also can metabolize glucose and mitigate sulfonamides through the co-metabolism of organic matter and sulfamethoxazole, contributing to antibiotic removal ​(Zheng et al., 2021)​. This unique metabolic capability further underscores the importance of *Pseudomonas* in environmental remediation and pollutant removal.

A slight increase in the abundance of *Pseudomonas* was observed in Site 3, which has plants nearby. Given that most pathogenic members of this genus are related to plants ​(Özen and Ussery, 2012)​, this relationship could be a pivotal factor in its distribution. The presence of plants and the specific environmental conditions at this site might have provided a competitive advantage for *Pseudomonas*, leading to its increased abundance.

On the other hand, the genus *Polynucleobacter* showed high abundance in the Abobral small lake and low abundance in the Aquidauana small lake, suggesting a higher susceptibility to pollution from the farm. Our results differ from a study that found a high abundance of *Polynucleobacter* in a river influenced by effluents from backyard aquacultures ​(Nakayama et al., 2017)​. It has also been found in polluted rivers and is known to live as chemoorganotrophs by utilizing low-molecular-weight substrates derived from the photooxidation of humic substances ​(Hahn et al., 2012; Ma et al., 2016)​.

Contrary to expectations, there was an increase in Bacteroidetes in the Aquidauana small lake. Although this phylum is more known for adapting well or even preferring polluted environments ​(Da Silva et al., 2015; Tai et al., 2020)​, pollution-sensitive Bacteroidetes have been observed, to function as bioindicators ​(Wolińska et al., 2017)​. This suggests that our environment might have conditions that are not unfavorable for this group.

The Actinobacteria phylum, which does not seem to be significantly influenced by the environmental conditions of the two regions studied, was found to be consistently present. Its genus, *Streptomyces*, was notably abundant across all four sites as well. This prevalence could be attributed to the fact that *Streptomyces* bacteria are the source of most antibiotics used in medicine, veterinary practice, and agriculture ​(Chater et al., 2010)​, making them efficient competitors in natural environments.

Site 3 showed a minor increase in *Streptomyces* abundance. This could be due to their close relationship with plants, as they are common in the rhizosphere and are frequent endophytes. Their ecological function in the natural decomposition of plant and fungi cell walls, which are globally abundant, could also contribute to this increase. *Streptomyces* are known for their significant role in breaking down plant, fungi, and insect cell walls or surface components ​(Chater et al., 2010)​.

On the other hand, the genera *Mycobacterium* and *Mycolicibacterium* of this phylum, demonstrated a different pattern. While their abundance remained relatively stable and low in Sites 1, 2, and 3, a significant peak was observed in Site 4. This pattern can be attributed to the natural resistance of *Mycobacterium* species to most antimicrobial agents currently available ​(Nguyen and Pieters, 2009)​.

The absence of plants in Site 4, which could help eliminate antibiotics, might have exposed this site to more antibiotics, favoring the growth of these genera. In particular, the genus *Mycolicibacterium*, which was recently separated from *Mycobacterium*, indicating its close evolutionary proximity, may share similar characteristics, such as a higher resistance to antibiotics ​(Gupta et al., 2019)​.

Cyanobacteria, a crucial component of many aquatic ecosystems, were found in significant amounts in all samples, demonstrating their resilience and adaptability in diverse environments. Despite being common in aquatic environments, they were found in greater relative abundance in Aquidauana compared to Abobral, likely due to the higher availability of nutrients such as phosphorus and nitrogen.

*Synechococcus*, a genus of cyanobacteria, exhibited a unique distribution pattern. Its abundance was low in the Abobral small lake, with no instances at Site 1. Conversely, in the Aquidauana small lake, there was a marked increase in its abundance. According to our data, this genus seems to be more sensitive to nutrient availability variations, as indicated by its differing abundance in Aquidauana and Abobral.

The presence of *Synechococcus* has been linked to total nitrogen, dissolved nitrogen, dissolved organic carbon, and dissolved phosphorus ​(Le et al., 2022)​. Furthermore, a study by Pishbin et al. (2021) found that under mixotrophic conditions, *Synechococcus elongatus* could remove up to 85.1% of phosphorus and 87.4% of nitrogen. This nutrient removal efficiency, coupled with the likely high levels of phosphorus and nitrogen in the Aquidauana small lake due to its location in a farming region, could account for the observed distribution pattern of *Synechococcus*, and cyanobacteria in general.

## 5 Conclusion

In this study, we collected samples from two different small lakes. The first small lake is located in Abobral sub-region, which is in a protected reserve area, and the second one is in Aquidauana sub-region, characterized by farming activities. For each of these locations, we collected samples from areas close to and far from the floating macrophyte *Eichornia crassipes*, giving us a total of four samples.

In our successful endeavor to unravel these two areas’ resistome, we were able to identify the primary antibiotic resistance genes and taxa, and how these vary depending on the level of pollution in the area and the presence or absence of plants nearby. We also identified a potential natural reservoir of the RPOB2 gene, as it occurred in high abundance in both areas. This finding is of academic, economic, and public health interest as it could influence decisions regarding the use of antibiotics in the area.

Our analysis of the collected data revealed a significant loss of both genetic and taxonomic biodiversity in the sample from the farm in Aquidauana sub-region when compared to the sample from the reserve in Abobral sub-region. This finding supports the widely accepted view that human activities can lead to a decrease in biodiversity.

However, our study also brought to light an interesting observation that contradicts a well-studied phenomenon. We found that human activity does not always result in an increase in the number of antibiotic resistance genes in the nearby bacterial community. This is one of the first studies to report such a finding, highlighting the need for further research in this area.

While the impact of human activity on the loss of genetic and taxonomic biodiversity is well-documented, our understanding of its effect on the diversity of resistance genes is still limited. More research is needed to investigate if human activity is causing a loss in diversity of resistance genes, and if so, whether certain genes are being favored over others. This will help us gain a more comprehensive understanding of the complex interactions between human activity and microbial communities.

Furthermore, we may have identified a potential natural reservoir for the RPOB2 gene, given its significant presence in both Aquidauana and Abobral. This discovery could facilitate informed decision-making regarding the use of antibiotics and public health.

## Data Availability

The raw sequences have been deposited in the NCBI Sequence Read Archive under the code PRJNA1078255.
