# Supplementary figures and images for: A comparative genomics study of the microbiome and freshwater resistome in Southern Pantanal
Source: Front Genet. 2024 Apr 18;15:1352801. doi: 10.3389/fgene.2024.1352801 (PMC11063290; doi:10.3389/fgene.2024.1352801)

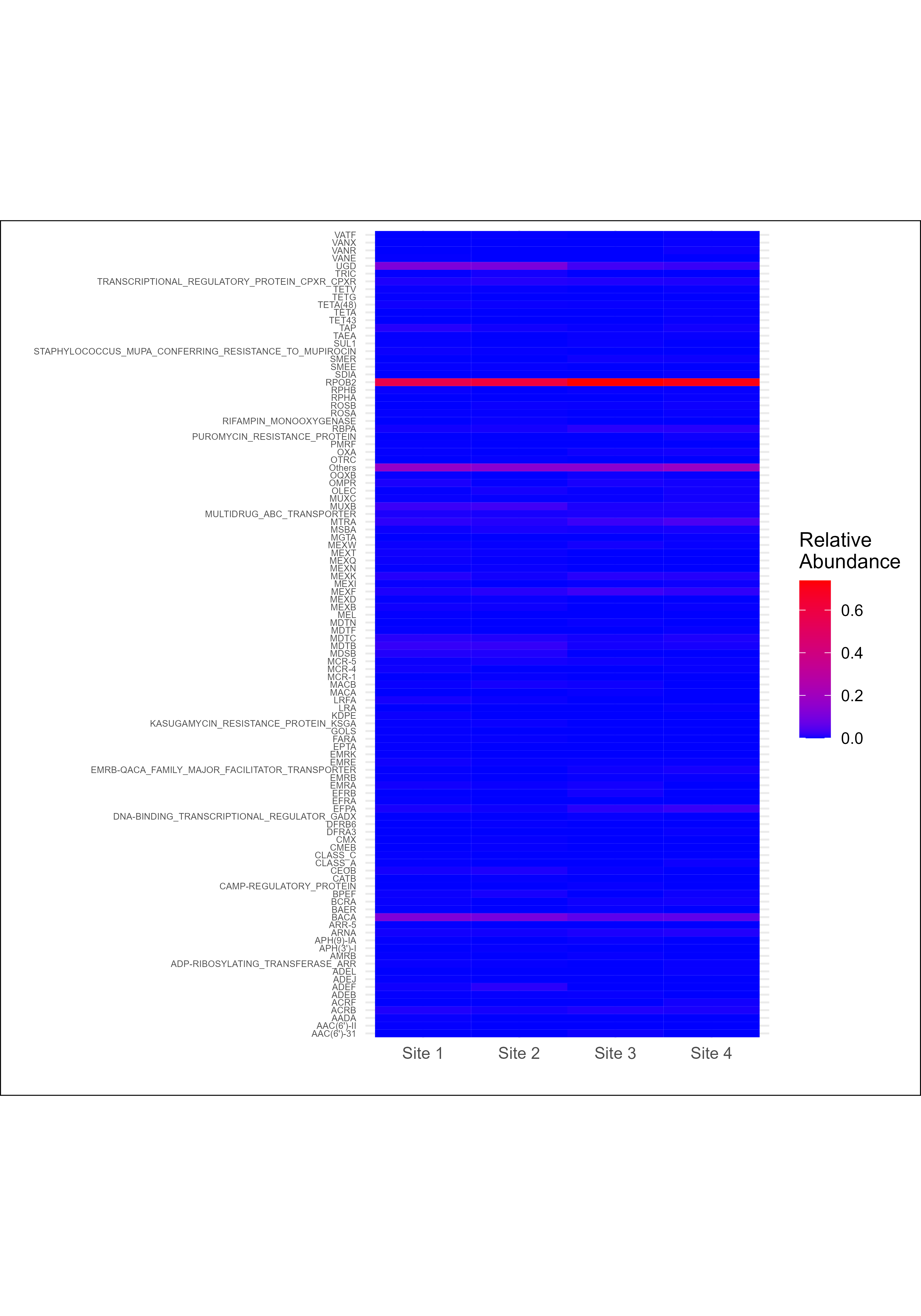

Supplement: Supplementary file 1 [file Image3.TIFF]

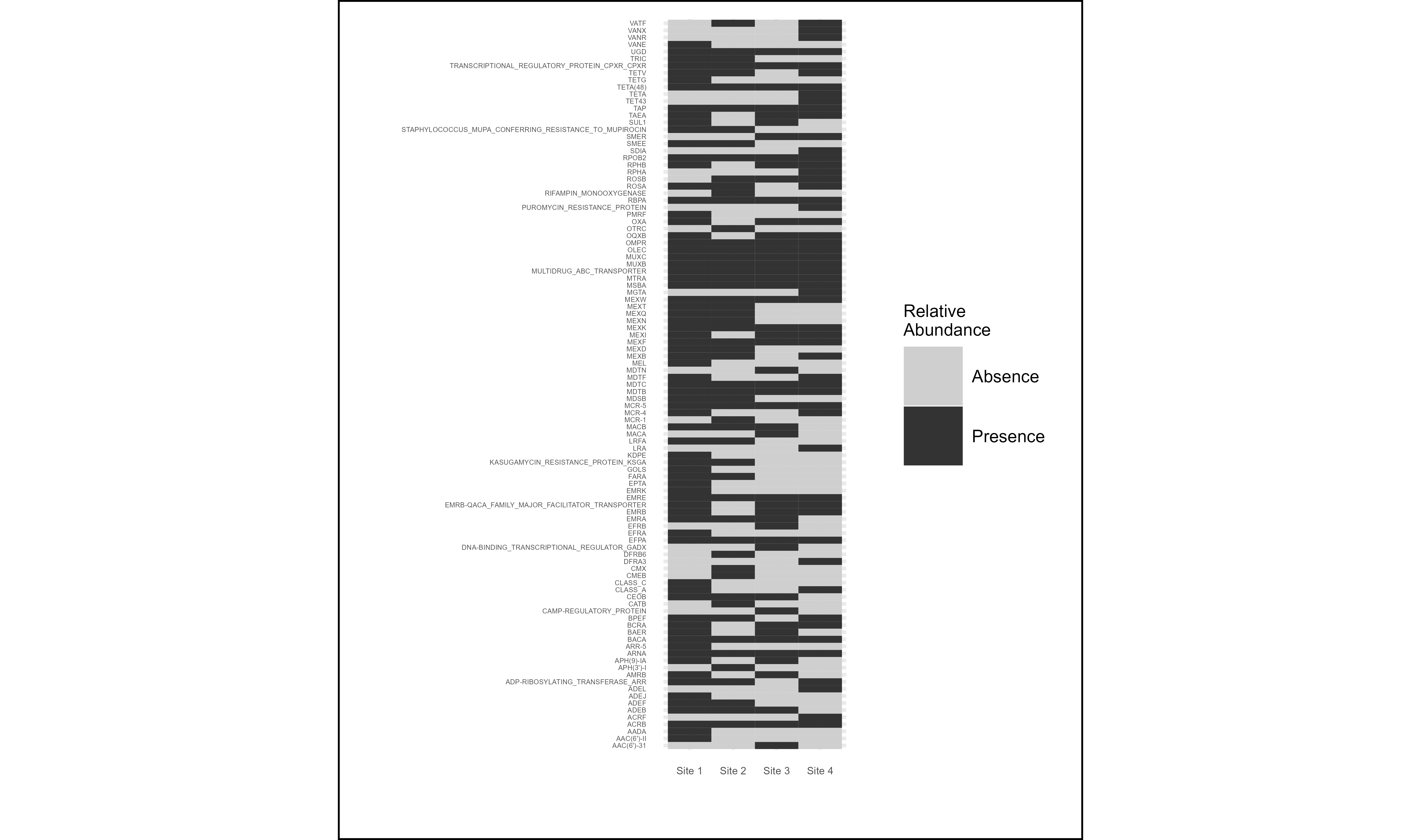

Supplement: Supplementary file 2 [file Image1.TIFF]

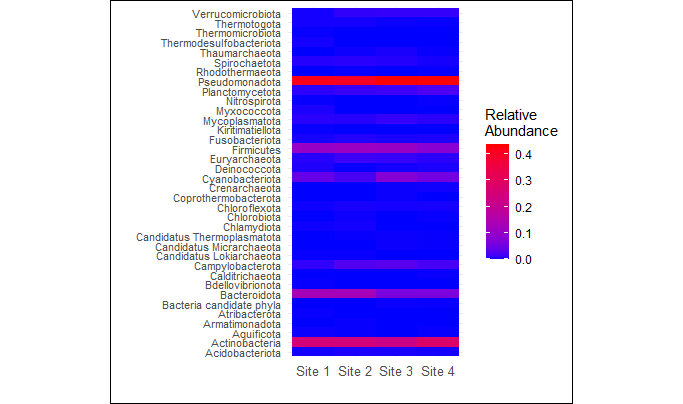

Supplement: Supplementary file 3 [file Image5.TIFF]

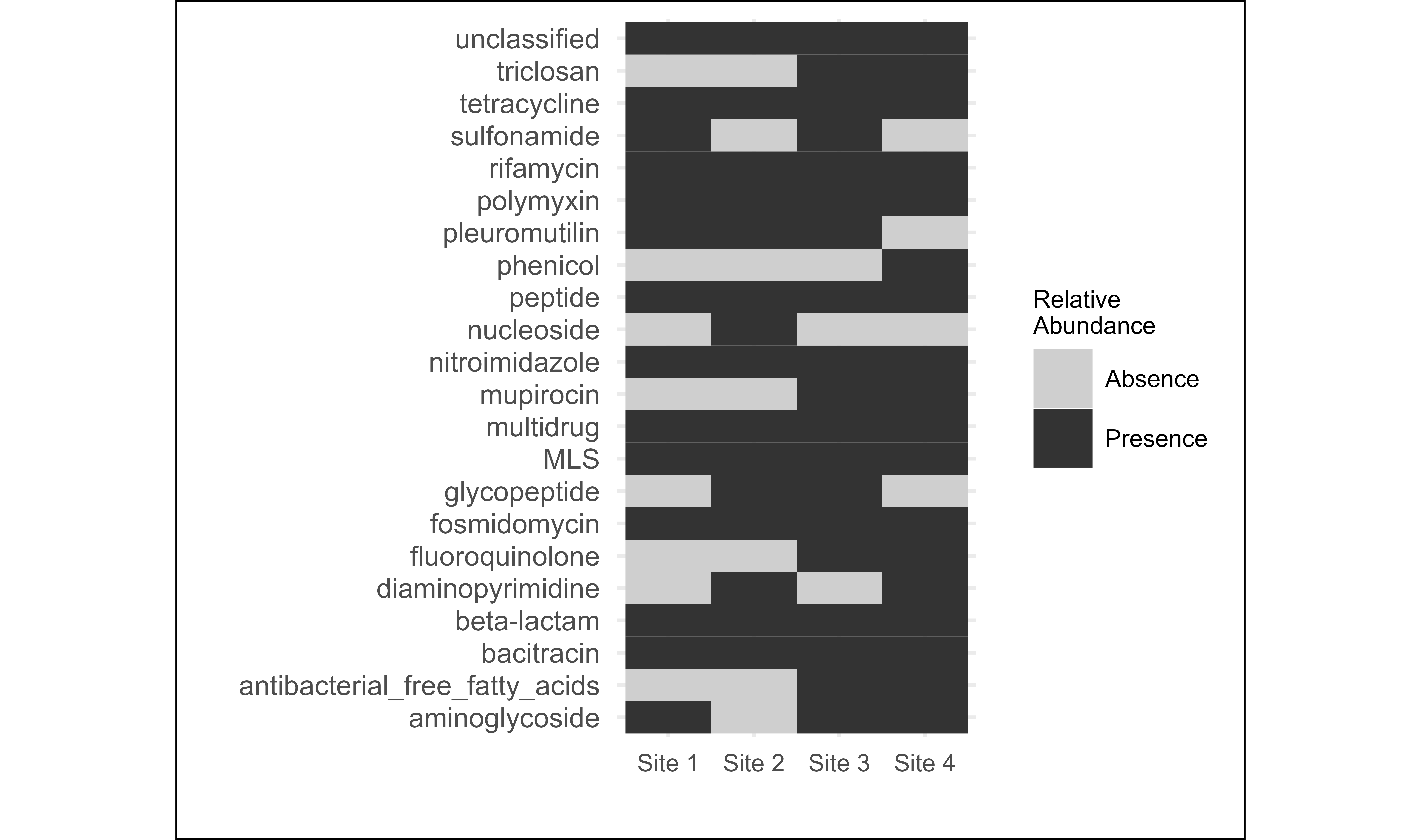

Supplement: Supplementary file 5 [file Image2.TIFF]

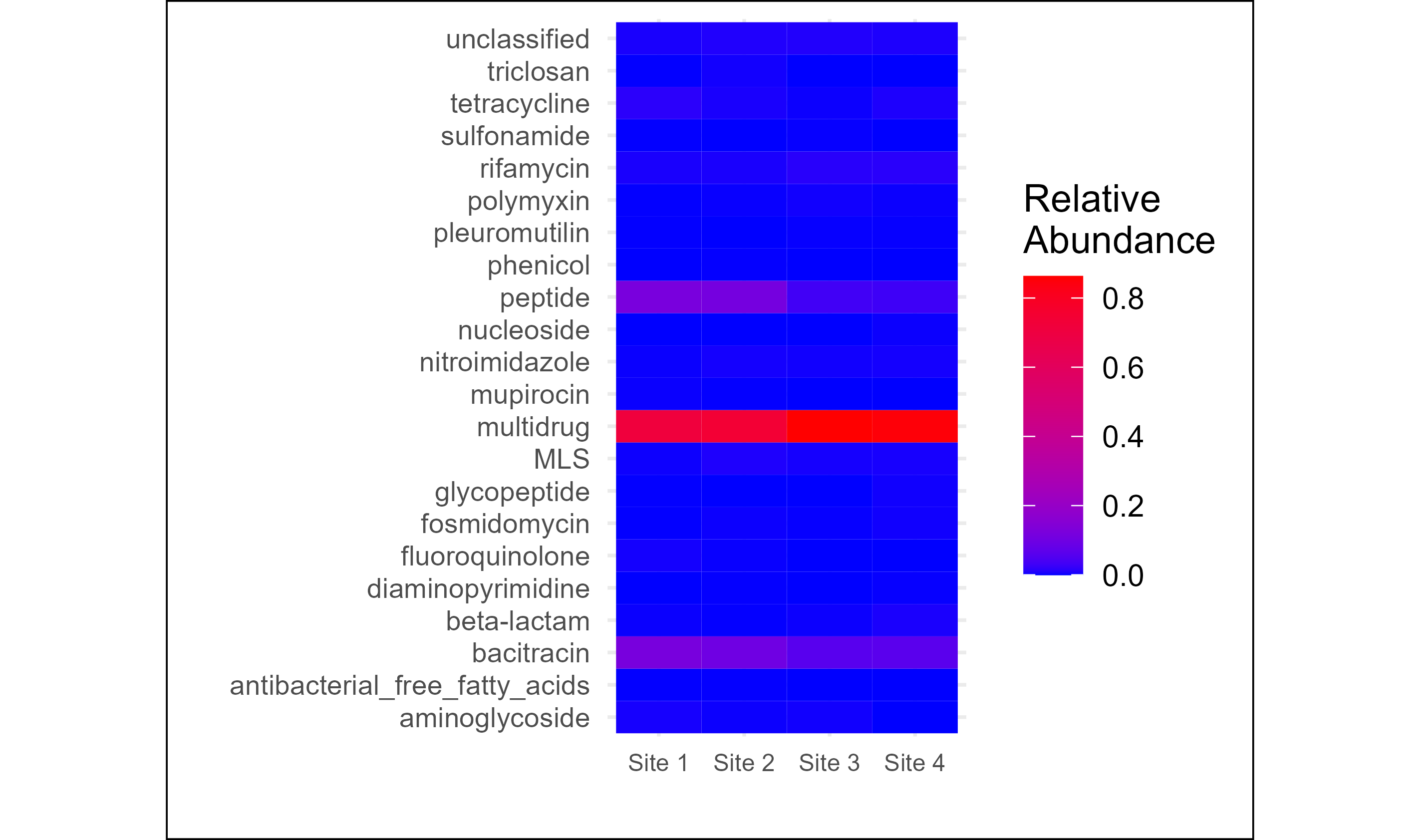

Supplement: Supplementary file 6 [file Image4.TIFF]
